# Contrasting Quiescent G_0_ Phase with Mitotic Cell Cycling in the Mouse Immune System

**DOI:** 10.1371/journal.pone.0073801

**Published:** 2013-09-16

**Authors:** Michio Tomura, Asako Sakaue-Sawano, Yoshiko Mori, Mitsuyo Takase-Utsugi, Akihiro Hata, Kenji Ohtawa, Osami Kanagawa, Atsushi Miyawaki

**Affiliations:** 1 Laboratory for Autoimmune Regulation, Research Center for Allergy and Immunology, RIKEN, Yokohama City, Kanagawa, Japan; 2 Laboratory for Cell Function and Dynamics, Brain Science Institute, RIKEN, Wako City, Saitama, Japan; 3 Center for Innovation in Immunoregulative Technology and Therapeutics, Kyoto University Graduate School of Medicine, Kyoto City, Japan; 4 Life Function and Dynamics, ERATO, JST, Wako City, Saitama, Japan; 5 Research Resource Center, Brain Science Institute, RIKEN, Wako City, Saitama, Japan; Innsbruck Medical University, Austria

## Abstract

A transgenic mouse line expressing Fucci (fluorescent ubiquitination-based cell-cycle indicator) probes allows us to monitor the cell cycle in the hematopoietic system. Two populations with high and low intensities of Fucci signals for Cdt1(30/120) accumulation were identified by FACS analysis, and these correspond to quiescent G_0_ and cycling G_1_ cells, respectively. We observed the transition of immune cells between quiescent and proliferative phases in lymphoid organs during differentiation and immune responses.

## Introduction

In addition to the four conventional phases of the cell cycle (G_1_, S, G_2_, and M), there is a fifth phase, G_0_, which denotes the nonproliferating or quiescent state of cells that have withdrawn from the active cell cycle [1], [2]. At a certain point during G_1_, a cell decides whether it will remain in G_1_ or retreat from the active cell cycle into G_0_.

We established the Fucci (fluorescent ubiquitination-based cell-cycle indicator) system to visualize cell-cycle progression in cultured cells and mouse embryos. This technique utilizes the ubiquitin oscillators that control cell cycle transitions [3], [4]. The probe consists of mKO2-hCdt1(30/120) and mAG-hGem(1/110), which function as G_1_(G_0_) and S/G_2_/M markers, respectively. These two chimeric proteins accumulate reciprocally in the nuclei of transfected mammalian cells, labeling nuclei of G_1_(G_0_) cells red (mKO2-positive) and S/G_2_/M cells green (mAG-positive). Using the CAG promoter [5], we generated transgenic mouse lines that express mKO2-hCdt1(30/120) (#596) or mAG-hGem(1/110) (#504). Using embryos of a cross-bred mouse line, #596/#504, described in our previous study, we performed time-lapse imaging of the cell cycle of neural progenitor cells during their migration and differentiation [3], [4].

Many cells in the adult animal body stay in G_0_. However, the regulation of the G_1_/G_0_ transition varies among different cell types. Whereas terminally differentiated cells, such as neurons and muscle cells, rarely divide, most lymphocytes are assumed to withdraw from and reenter the cell cycle repeatedly throughout their lifetime. We thus planned to study dynamic transition between quiescence and proliferation of lymphocytes using Fucci transgenic mice. Although the line #596/#504 has been useful for studying relationships between cell-cycle progression and morphogenesis in many organs, we noticed that neither mKO2-hCdt1(30/120) nor mAG-hGem(1/110) was expressed in the hematopoietic system of this line. Thus, we screened a pool of Fucci transgenic mouse lines constructed with the CAG promoter, and found that #639 and #474 exhibit hematopoietic gene expression of mKO2-hCdt1(30/120) and mAG-hGem(1/110), respectively. We then investigated Fucci signals in immune cells from these two lines, which are hereafter referred to as FucciG_1_-#639 and FucciS/G_2_/M-#474.

## Materials and Methods

### Ethics Statement

The experimental procedures and housing conditions for animals were approved by the Animal Experimental Committees at the Institutes of Physical and Chemical Research (RIKEN) -Research Center for Allergy and Immunology (RCAI) and -Brain Science Institute (BSI), and Kyoto University school of medicine, and all animals were cared for and treated humanely in accordance with the Institutional Guidelines for Experiments using Animals.

### Mice

FucciG_1_-#639 and FucciS/G_2_/M-#474 mice of BDF1 background were generated as described previously [3]. These transgenic mice were backcrossed to C57BL/6J mice (CREA Japan Inc.) more than three times and crossmated, then the resulting progeny, FucciG_1_-#639/FucciS/G_2_/M-#474 double transgenic mice (#639/#474 mice) were used for experiments.

### Cell Culture and Imaging

NMuMG/Fucci cells were grown in DMEM (high glucose) supplemented with 10% fetal bovine serum (FBS), penicillin/streptomycin, and 10 µg/ml insulin (Sigma: I0516). Cells were fixed with 1% PFA for 1 hour at room temperature and then with 70% ethanol for overnight. This procedure was sufficient for effective fixation while avoiding the quencing of fluorescent proteins. After being washed, cells were stained with Alexa Fluor 647-conjugated anti-Ki-67 monoclonal antibody (mAb) (BD Pharmingen) and DAPI, then analyzed using a FACSAria II (BD Biosciences). Data were analyzed using FlowJo software (Tree star). Time-lapse imaging and data analysis were performed as described previously [3].

### Stimulation of Immune Cells

Splenocytes (1×10^7^ cells/10 ml) were stimulated with concanavalin A (ConA) (Sigma) (5 µg/ml) plus IL-2 (200 U/mL) or lipopolysaccharides (LPS) (Sigma) (10 µg/ml) for 2 days, for stimulation of T and B cells, respectively.

### Flow Cytometry Analysis

Antibodies used in this study were purchased from BD Pharmingen, eBioScience, or BioLegend. After being washed with Dulbecco’s phosphate-buffered saline (PBS) containing 2% fetal calf serum (FCS) and 0.02% sodium azide (staining buffer), cells were treated with culture supernatant from the 2.4G2 hybridoma for blocking Fc binding, and subsequently with appropriate fluorochrome-conjugated antibodies. For T and B cell gating, splenocytes were stanined with allophycocyanin (APC)-conjuagted anti-CD3 mAb and APC-conjuagted anti-CD19 mAb, respectively. Thymocytes were stained with APC-conjuagted anti-CD3 mAb, APC-Cy7-conjuagted anti-CD8 mAb, and Pacific blue-conjugated anti-CD4 mAb. For gating differentiating B cell populations, bone marrow (BM) cells were stained with PE-Cy7- or APC-conjugated anti-IgM mAb, biotinylated anti-B220, and Pacific blue-conjugated anti-CD43 mAb, followed by Pacific orange-conjugated streptavidin (Invitrogen). For detection of Ki-67, cells were treated with anti-surface antigen antibodies and fixed with 1% PFA at 4° C for 15 min, followed by 75% ethanol at −20°C for 2 hours, then stained with Alexa 647-conjugated anti-Ki-67 mAb or Alexa 647-conjugated control mAb. Stained cells were analyzed using a FACS Calibur (BD Biosciences) and a Fortessa (BD Biosciences). mAG and mKO2 signals were detected via the FITC and PE channels, respectively. Flow cytometry data were analyzed using the FlowJo software (Tree Star, Inc.).

### Cell Sorting and Stimulation

Cell suspensions prepared from spleen and lymph node (LN) of #639/#474 mice were treated with culture supernatant from the 2.4G2 hybridoma, stained with APC-conjuagted anti-CD5 mAb for gating T cells or APC-Cy7-conjugated anti-CD19 mAb for gating B cells. Then, CD5^+^mKO2^++^mAG^−^ cells or CD19^+^mKO2^++^ mAG^−^ cells were sorted by FACS Aria III (BD Biosciences) with purity of >99%. Collected CD5^+^mKO2^++^mAG^−^ cells (1×10^6^/1 ml/well) or CD19^+^mKO2^++^mAG^−^ cells (1×10^6^/1 ml/well) were plated in 48-well plates and cultured in the presence of ConA (5 µg/ml) or LPS (10 µg/ml), respectively.

### Intravital Microscopy

Inguinal LNs dissected from #639/#474 mice were used for imaging resting lymphocytes. For imaging of draining LNs after immunization, a #639/#474 mouse was injected subcutaneously in the rear footpad with 20 µl of CFA (DIFCO) emulsion with 20 µg of TNP (17)-KLH (Keyhole Limpet Hemocyanin) (BIORESEARCH TECNOLOGIES, Inc.). Three days later, draining popliteal LNs were dissected, and maintained at 37°C under a superfused medium bubbled with 95% O2/5% CO2. Signals of mKO2, mAG, and second harmonic generation (SHG) were obtained by a femto-second pulsed laser at 930 nm (Chameleon vision II, Coherent Inc.). These signals were collected using 3 channels, which were separated using 495, 560, and 593 nm dichroic mirrors in combination with 492SP (SHG), 525/50 (mAG), and 575/25 (mKO2) and 629/56 (mKO2) nm band-pass filters. A 25× water-immersion objective (N.A. = 1.1) and GaAs non-descanned detectors (A1R MP, Nikon) were used.

### Distribution of Materials

Transgenic mouse lines, FucciG_1_-#639 and FucciS/G_2_/M-#474, will be distributed from the RIKEN Bio-Resource center experimental animal division (http://www.brc.riken.go.jp/lab/animal/en/).

## Results and Discussion

**Figure 1:**
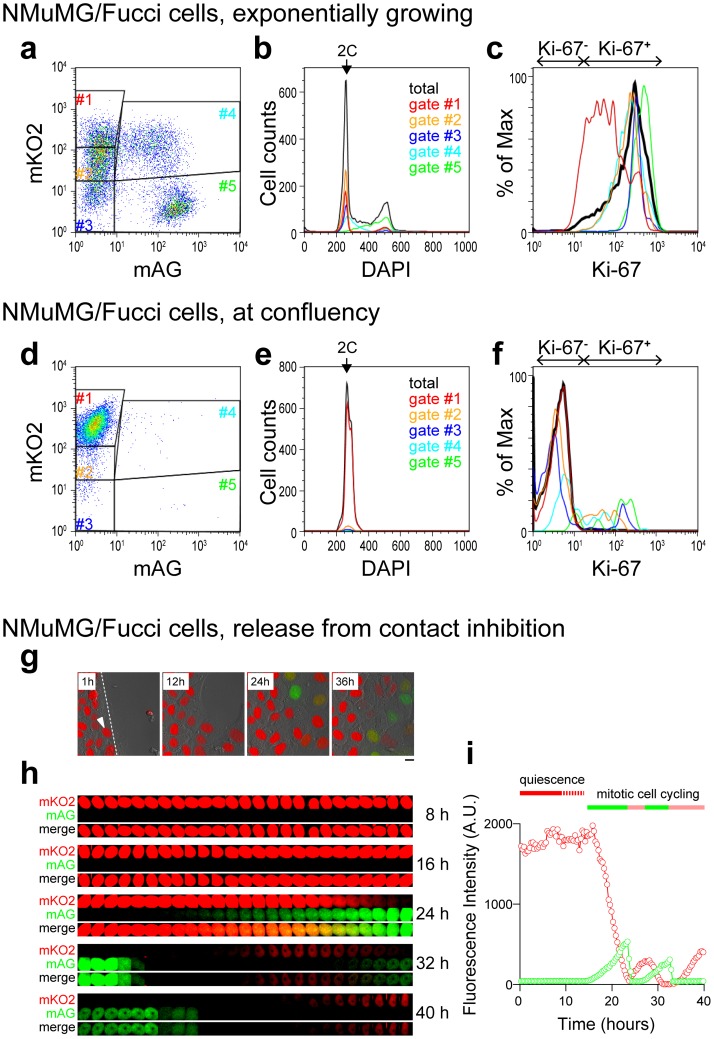
Characterization of Fucci-expressing NMuMG cells. (**a–f**) Flow cytometry analysis of Ki-67- and DAPI-stained NMuMG/Fucci cells. Fucci signals (mKO2 vs. mAG) of exponentially growing cells (**a**) and cells at confluency (**d**). Cells collected in gate #1 were found to be diploid (2 C) and Ki-67 negative, and thus were assigned to quiescent G_0_ phase. (**g**) Fluorescence images of NMuMG/Fucci cells, after a “wound” (scratch) was introduced into a confluent monolayer. Scale bar, 10 µm. (**h, i**) Time-lapse imaging (**h**) and fluorescence intensity (**i**) of mKO2-hCdt1(30/120) (red line) and mAG-hGem(1/110) (green line) in the cell indicated by the white arrowhead in (**g**).

To examine quiescence-associated cell-cycle dynamics in cultured cells, we used Fucci-expressing, stably transformed normal murine mammary gland (NMuMG) cells (NMuMG/Fucci cells), which show confluence-induced proliferation arrest [3], [4]. We analyzed their Fucci signals and Ki-67 immunosignals when cells were exponentially growing (**Fig. 1a–c**) and after they reached confluency (**Fig. 1d–f**). We gated 5 populations accroding to the intensities of mKO2 and mAG (**Fig. 1a, d**). Gates #2 through #5 in growing condition showed Ki-67 positive signals and should represent the mitotic cell cycling phases. Gate #1 contained cells that showed an order of magnitude lower Ki-67 signal than the other gated cells. Remarkably, nearly all of the cells at confluency were collected in gate #1. These cells contained bright red (mKO2^++^/mAG^−^) (**Fig. 1d**), diploid (**Fig. 1e**), and Ki-67 negative (**Fig. 1f**) cells, which were supposed to stay in quiescent G_0_ phase.

After all cells showed bright red nuclei (mKO2^++^/mAG^−^, quiescent G_0_ phase), a wound was introduced mechanically; cells at the edge of the wound turned green after a latency period of 12–20 hr (**Fig. 1g** **, and Movie S1**), reminiscent of the time (8 hr) required for NIH 3T3 cells to reenter the cell cycle from the G_0_ state after the onset of proliferation stimuli [6]. The latency time we observed should consist of the 8 hr required to re-enter the cell cycle, plus the time required for cells to proceed through the remainder of G_1_. One cell (indicated by an arrowhead in **Fig. 1g**) was tracked for 40 hr (**Fig. 1h**). Temporal profiles of the intensities of red and green signals in this cell are shown in **Fig. 1i**. It was apparent that the red fluorescence in the G_0_ state was several times stronger than in the cycling G_1_ state. The differential intensity of red fluorescence between quiescent and proliferating cells was observed previously in the developing cerebral cortex of #596/#504 mice; postmitotic neurons in the cortical plate exhibited much brighter red fluorescence than mitotic neural progenitors in the ventricular zone, presumably due to accumulation of mKO2-hCdt1(30/120) after cell-cycle exit [3], [4].

**Figure 2:**
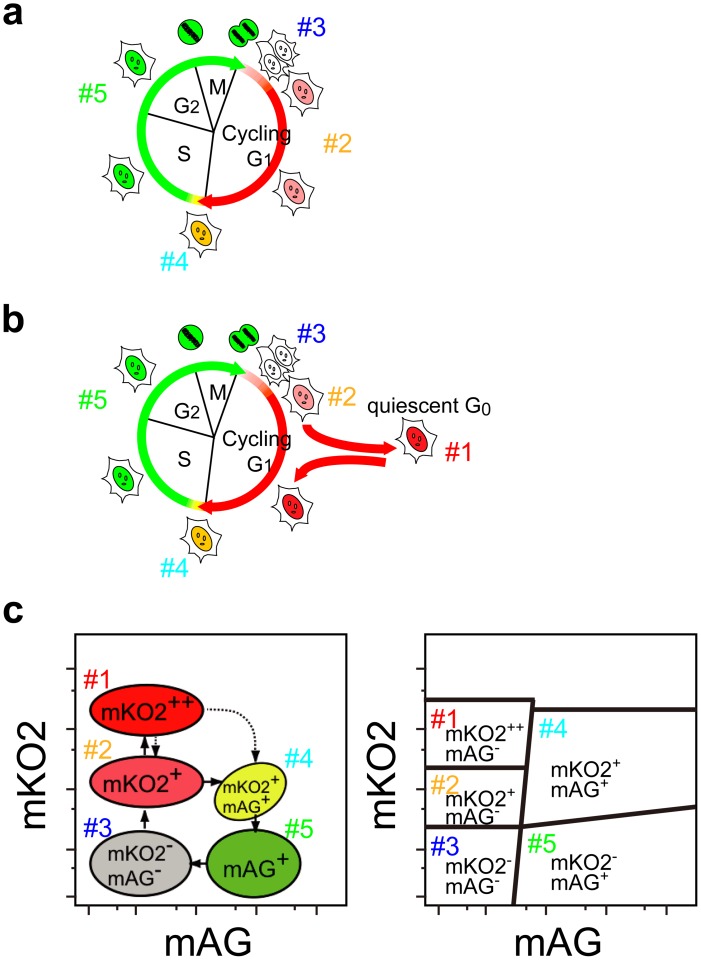
Quiescence-associated cell-cycle dynamics and Fucci signals. (**a, b**) mKO2-hCdt1(30/120) and mAG-hGem(1/110) fluorescent probes label individual G_1_ phase nuclei (mKO2-positive) and S/G_2_/M phase nuclei (mAG-positive). (**a**) Cells in G_1_ phase during continuous cell division (cycling G_1_ cells) go into S/G_2_/M phase (gates #4 and #5) and express mAG before reaching mKO2 level comparable to those of non-dividing cells. (**b**) mKO2-positive cells can be subdivided into cycling G_1_ (gates #3 and #2) and quiescent G_0_ (gate #1). Accumulation of mKO2-hCdt1(30/120) continues after cell-cycle exit; subsequently, quiescent G_0_ cells exhibit high levels of mKO2-signal. (**c**) Schematized 5 populations gated according to Fucci signals. Note that cells in G_0_ exhibit the highest level of mKO2 signal (mKO2^++^ cells); cells in G_1_ exhibit lower mKO2 levels than cells in G_0_ (mKO2^+^ cells).

Since the cumulative red signal of mKO2-hCdt1(30/120) barely reached its saturation level, we concluded that it would be possible to discriminate cells that have entered G_0_ from cells staying in G_1_ by quantifying the intensity of mKO2 fluorescence (**Fig. 2a, b**). The five gated populations in terms of Fucci signals are schematized in **Figure 2c**; mKO2^++^/mAG^−^ (gate #1), mKO2^+^/mAG^−^ (gate #2), mKO2^−^/mAG^−^ (gate #3), mKO2^+^/mAG^+^ (gate #4), and mKO2^−^/mAG^+^ (gate #5) correspond to cells in G_0_, G_1_, very early G_1_, G_1_/S, and S/G_2_/M, respectively (**Fig. 2c**).

**Figure 3:**
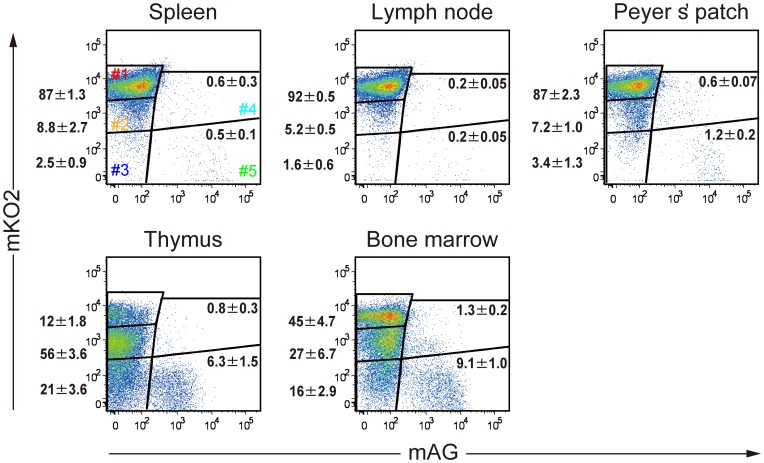
Flow cytometric profiles of Fucci signals in immune cells from various organs of #639/#474 mice. Single-cell suspensions from spleen, lymph node, Peyer’s patch, thymus, and bone marrow of #639/#474 mice were prepared and subjected to flow-cytometric analysis in order to analyze the levels of mAG and mKO2 signals. Data are representative of three individual experiments. Numbers in dot plots indicate mean ± S.D. of gated cells.

We aimed to analyze both quiescence and proliferation of lymphocytes using Fucci transgenic mice. We crossbred FucciG_1_-#639 and FucciS/G_2_/M-#474 to generate double transgenic mice (#639/#474 mice) for analyzing Fucci-signals of immune cells in various lymphoid organs (**Fig. 3**). Bone marrow (BM) and thymus, as well as secondary lymphoid organs, such as lymph node (LN), spleen, and Peyer’s patch (PP), were isolated and analyzed with regard to mKO2 and mAG fluorescence intensities. Most lymphocytes in spleen, LN, and PP exhibited very strong mKO2 signal but no mAG signal, which should represent the mKO2^++^/mAG^−^ population (gate #1, G_0_ population). By contrast, cells in thymus and BM showed substantial heterogeneity; these cells can also be grouped into five types as illustrated in **Fig. 2c**. While NMuMG/Fucci cells show a high clonality, lymphocytes from #639/#474 mice are heterogenous in terms of gene expression of both mKO2-hCdt1(30/120) and mAG-hGem(1/110). Thus, it is noted that the lymphocytes collected in gate #3 should contain the ones that failed to synthesize either mKO2-hCdt1(30/120) or mAG-hGem(1/110).

**Figure 4:**
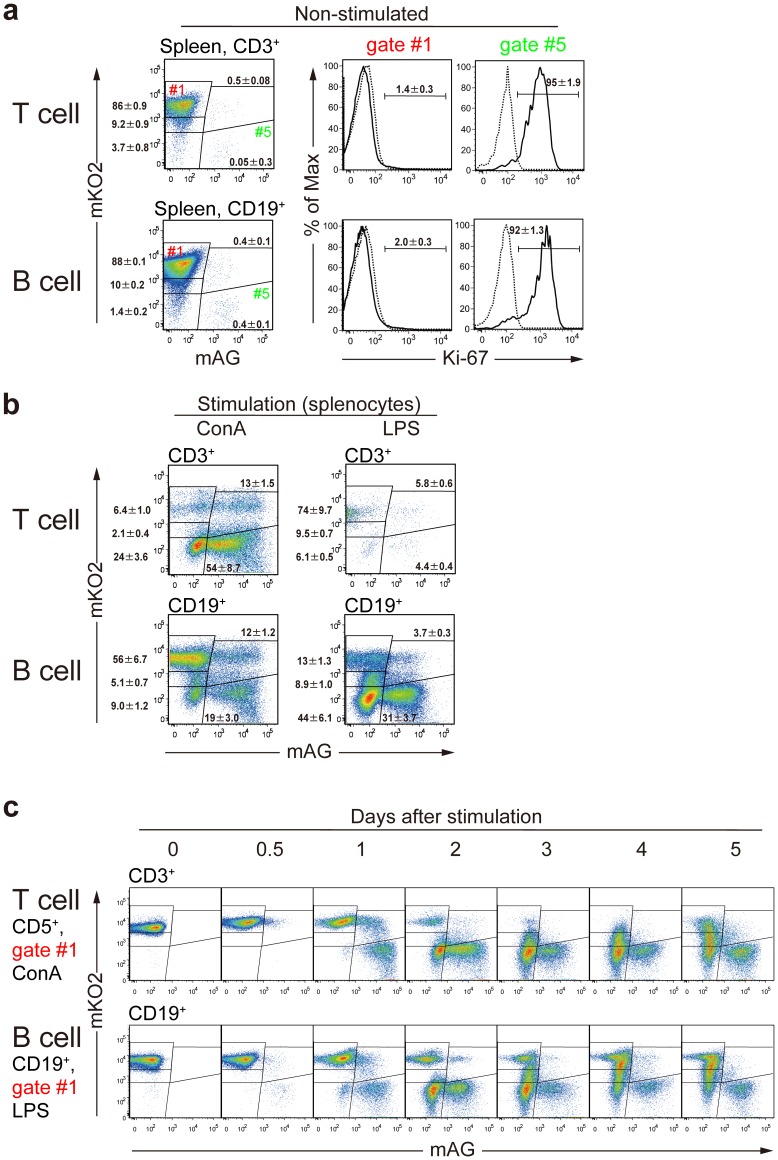
Stimulation-induced changes in Fucci signals in various immune cell subsets isolated from #639/#474 mice. (**a**) Splenocytes from #639/#474 mice were stained with APC-anti-CD3 mAb or APC-anti-CD19 mAb. Using flow cytometry, non-stimulated T and B cells were collected as CD3^+^ and CD19^+^ fractions, respectively. The cells were fixed and stained with Alexa 647-conjugated anti-Ki-67 mAb (solid line) and Alexa 647-conjugated control mAb (dotted line). The signals of Alexa 647 of cells in gate #1 and gate #5 are shown. (**b**) Cultured splenocytes were stimulated for 2 days with ConA and IL-2 or LPS, and T cells and B cells were gated as CD3^+^ and CD19^+^ cells, respectively. Data are representative of three individual experiments. Numbers in dot plots and histograms indicate mean ± S.D. of gated cells. (**c**) Sorted mKO2^++^ T cells (CD5^+^, gate #1) or B cells (CD19^+^, gate #1) were cultured with ConA or LPS, and Fucci-signals were analyzed at indicated time points. Data are representative of two individual experiments.

Next, we studied how stimuli caused cell-cycle re-entry in splenocytes, which are mostly composed of mature T and B lymphocytes staying in G_0_ (**Fig. 3**). Without stimulation, the majority of both T and B cells of splenocytes were collected in gate #1, which showed mKO2^++^/mAG^−^ and Ki-67(−) (**Fig. 4a**). After stimulation with ConA (**Fig. 4b**, left), a significant fraction of T cells became proliferative; proliferation was induced also in B cells but to a lesser extent. In contrast, stimulation with LPS induced proliferation only in B cells (**Fig. 4b**, right). We also observed temporal profiles of Fucci-signals after stimulation with ConA in T cells (CD5^+^, gate #1) (**Fig. 4c** **,** top) and LPS in B cells (CD19^+^, gate #1) (**Fig. 4c** **,** bottom). For both cell types, as the mKO2^++^/mAG^−^ population (gate #1) decreased, both the mKO^+^/mAG^+^ population (gate #4) and the mKO^−/^mAG^+^ population (gate #5) increased transiently; then, the mKO2^+^/mAG^−^ (gate #2) and the mKO2^++^/mAG^−^ (gate #1) populations recovered.

**Figure 5:**
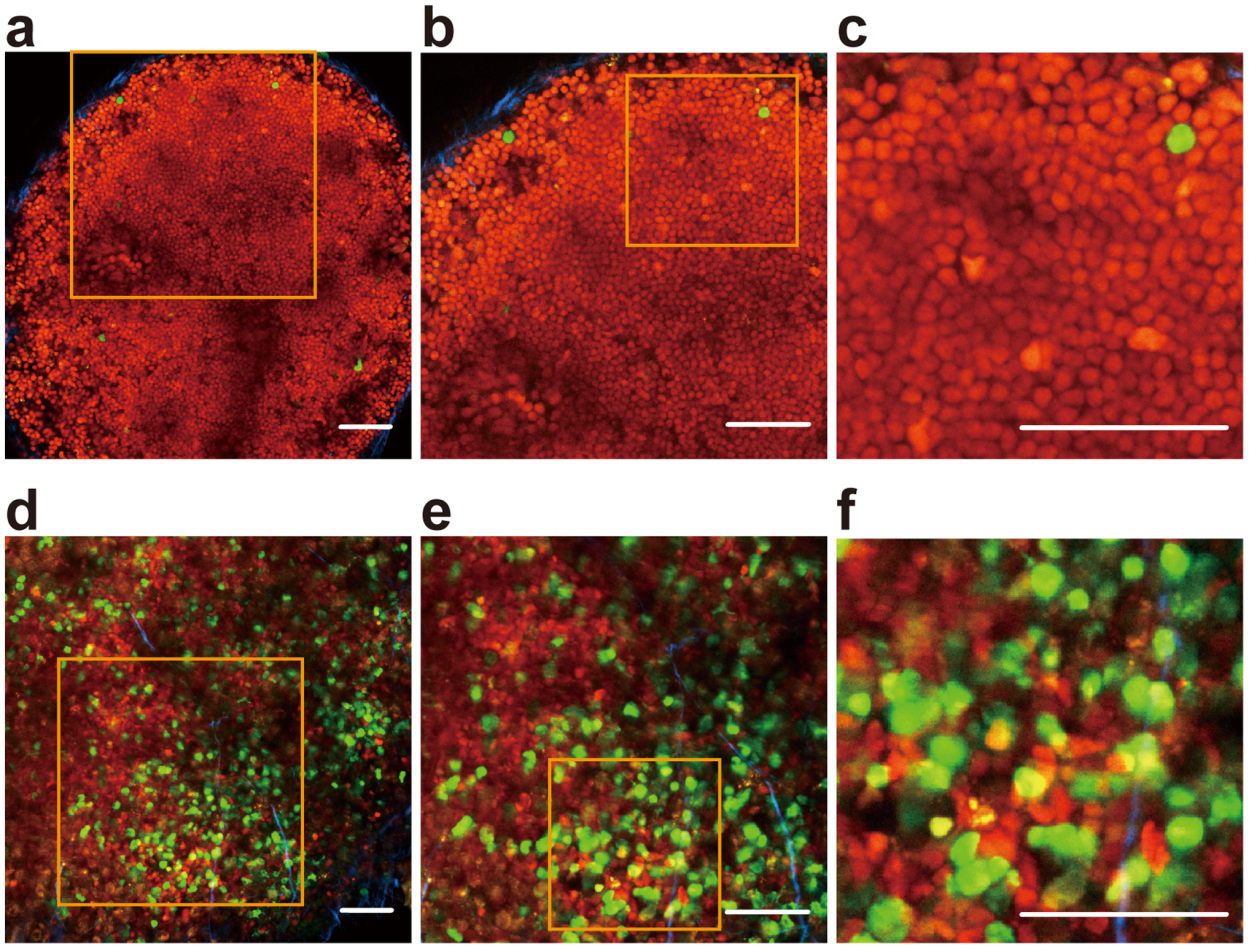
Visualization of Fucci-signals of lymph node cells in #639/#474 mice. (**a–c**) An inguinal LN was dissected and maintained at 37°C under superfused medium bubbled with 95% O_2_/5% CO_2_ and observed by 2-photon microscopy. Zoomed in on sequentially from (**a**) to (**c**). Orange squares in (**a**) and (**b**) are zoomed up in (**b**) and (**c**), respectively. (**d-f**) A popliteal LN 3 days post-innoculation of CFA emulsion with TNP (17)-KLH in the rear footpad. The LN was dissected and maintained at 37°C under superfused medium bubbled with 95% O_2_/5% CO_2_ and observed for 30 minutes. Zoomed in on sequentially from (**d**) to (**f**). Scale bars, 50 µm.

We then examined cell-cycle re-entry of lymphocytes in intact lymph nodes (LNs) of #639/#474 mice by two-photon excitation microscopy. On the one hand, intact inguinal LNs of #639/#474 mice were observed to be mostly filled with lymphocytes that contained bright red nuclei (**Fig. 5a**–**c**), confirming that most lymphocytes in an LN remain in G_0_ (**Fig. 3**). On the other hand, after the rear footpad of a #639/#474 mouse was injected subcutaneously with CFA/TNP-KLH, a considerable number of cells with green nuclei emerged in the popliteal draining LN (**Fig. 5d**–**f** and **Movie S2, S3**).

**Figure 6:**
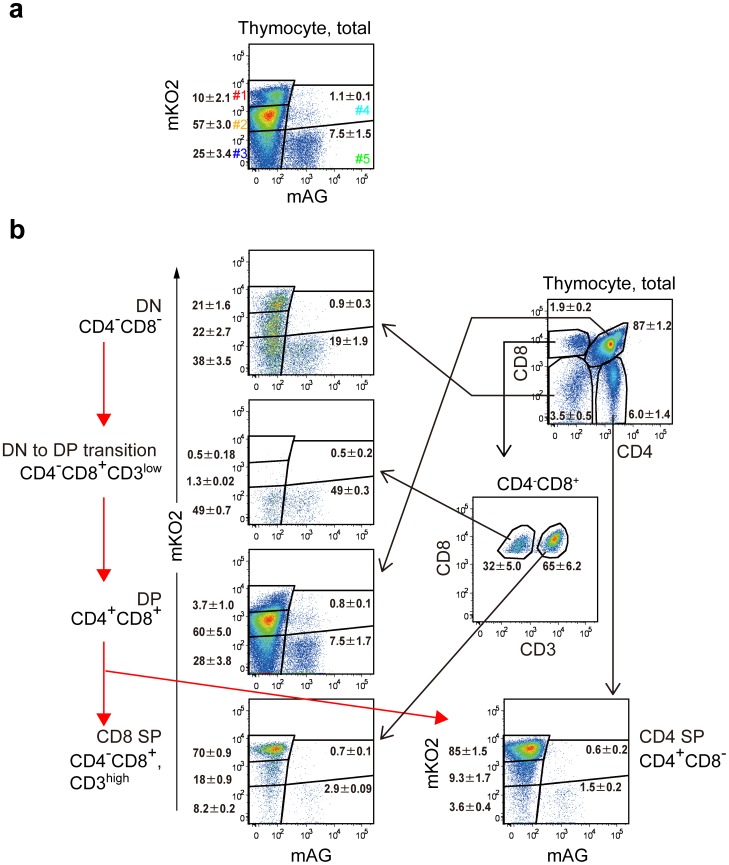
Flow cytometric profiles of Fucci signals of differentiating T cell subsets in thymus of #639/#474 mice. Thymocytes were stained with fluorochrome-conjugated anti-CD3, anti-CD4 and anti-CD8 mAbs, and subjected to flow cytometry. Total thymocytes were divided into CD4^−^CD8^−^ cells (DN), CD4^−^CD8^+^CD3^low^ cells (DN to DP transition), CD4^+^CD8^+^ cells (DP), CD4^−^CD8^+^CD3^high^ cells (CD8 SP) cells and CD4^+^CD8^−^ cells (CD4 SP), and levels of mAG and mKO2 signals in each subset are shown. Data are representative of three individual experiments. Numbers in dot plots indicate mean ± S.D. of gated cells.

Heterogeneity of Fucci signals in thymocytes (**Fig. 3** **,** **6a**) inspired us to analyze cell-cycle status during their differentiation in thymus. Immature CD4 CD8 double negative (DN) thymocytes differentiate to CD4 CD8 double positive (DP) thymocytes via CD8^+^CD3^low^ (DN to DP transition cells) cells. Then the DP thymocytes differentiate to CD4 SP thymocytes or CD8 SP thymocytes [7]–[9]. As the signal for CD3 is upregulated along with the thymocyte differentiation, the mature CD8 SP thymocytes were characterized as CD8^+^CD3^high^ and distinguished from the DN to DP transition cells (CD8^+^CD3^low^) cells. All these thymocyte populations were prepared and analyzed for the Fucci signals (**Fig. 6b**). While the most immature DN thymocytes were distributed evenly among the 5 gates, the terminally differentiated thymocytes were collected principally in gate #1. Interestingly, no accumulation of mKO2 signal was observed in DN to DP transition thymocytes, which may suggest very rapid cell-cycling of the immature thymocytes.

**Figure 7:**
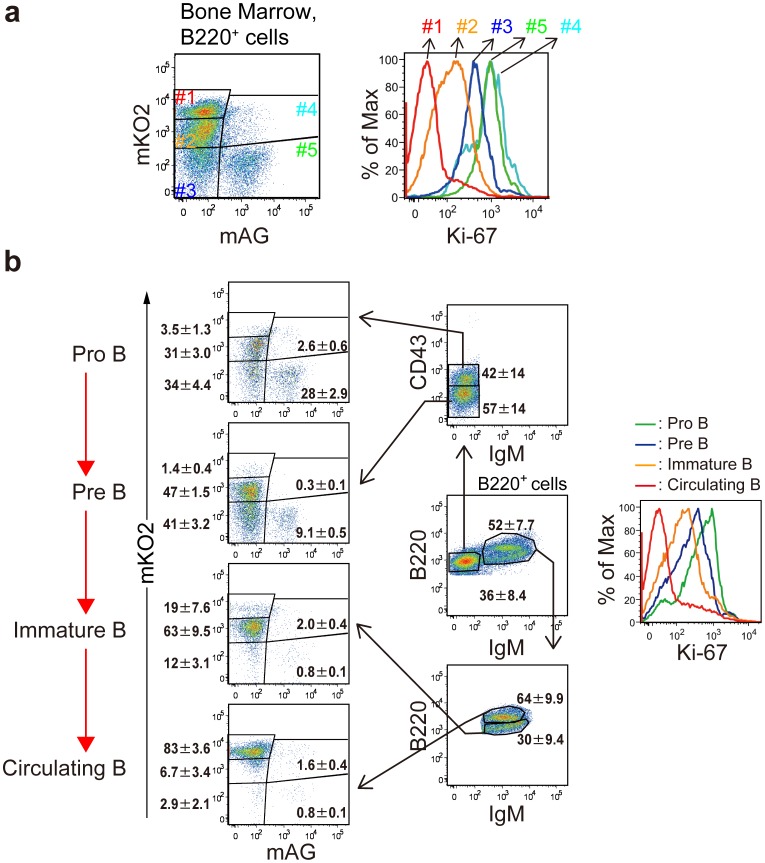
Flow cytometric profiles of Fucci signals of differentiating B cell subsets in bone marrow of #639/#474 mice. (**a**) BM cells were stained with PE-Cy7-conjugated anti-B220 mAb followd by Alexa 647-conjugated anti-Ki-67 or contorol mAbs. B220^+^ cells were divided into 5 populations based on the Fucci-signals (Left). Signals of Alexa 647-conjugated anti-Ki-67 mAb (solid line with the same color) are shown (Right). (**b**) BM cells were stained with fluorochrome-conjuagated anti-B220, anti-IgM, and anti-CD43 mAbs for flow cytometry. B220^+^ cells were divided into pro-B cells as B220^low^IgM^−^CD43^+^, pre-B cells as B220^low^IgM^−^CD43^−^ cells, immature B cells as IgM^+^B220^low^ cells and circulating B cells as IgM^+^B220^high^ cells. Levels of mAG and mKO2 signals in each subset are shown. Data are representative of three individual experiments. Numbers in dot plots indicate mean ± S.D. of gated cells.

Likewise, we investigated Fucci-signals during B cell differentiation in BM [10]–[12] (**Fig. 7**). A large number of B220^+^ cells in BM were mKO2^++^/mAG^−^ (gate #1) with the least Ki-67 expression, and thus considered to stay in G_0_ phase (**Fig. 7a**). BM cells were fractionated into Pro-B, Pre-B, Immature B, and mature circulating B cells according to intensities of signals for B220, IgM, and CD43 (**Fig. 7b**). Pro-B and Pre-B cell containing fractions showed heterogenous signals for mKO2^+^/mAG^−^ (gate #2), mKO2^+^/mAG^+^ (gate #4), mKO2^−/^mAG^+^ (gate #5), and mKO2^−/^mAG^−^ (gate #3), suggesting that these cell types were actively cycling. However, as Pre-B cells differentiated towards immature B cells, mAG intensity decreased and mKO2 intensity increased close to mature circulating B cells, which indicated its highest level (mKO2^++^) (**Fig. 7b**). We also confirmed that the B cell differentiation was correlated with decrease in Ki-67 expression (**Fig. 7b**).

Unlike neurons and skeletal muscle cells that exist in a terminally differentiated G_0_ state, most lymphocytes withdraw from the cell cycle only transiently and reenter the cycle in response to various stimuli. It is important to directly monitor the dynamic transition between cycling G_1_ and quiescent G_0_ cells. The Fucci technique is based on the cell-cycle−dependent degradation of proteins; thus, the signal intensity may be proportional to the length of the respective cell-cycle phase. There is evidence that transcription driven by the CAG promoter occurs in Ki-67-negative cells [13], which are assumed to be in G_0_ [5], [14]. Thus, mKO2-hCdt1(30/120) may gradually accumulate in G_0_ cells. In this study, we demonstrate that the intensity profile of the G_1_(G_0_) marker signal (the mKO2 fluorescence) exhibits two peaks: mKO2^++^ and mKO2^+^, which reflect quiescent G_0_ cells and cycling G_1_ cells, respectively. Here, we propose that FucciG_1_-#639 and/or FucciS/G_2_/M-#474 transgenic mice can provide reliable readouts of the cell-cycle regulation of lymphocytes, both *in vitro* and *in vivo*.

## Supporting Information

Movie S1 **Time-lapse imaging of Fucci-expressing NMuMG cells response to wound.** Fucci-expressing NMuMG cells were grown on a glass-bottom dish to reach the state of confluent (contact inhibition). One hour after a scratch of the monolayer cells, time-lapse imaging was performed using an LCV100 (Olympus). Images were acquired every 19 minutes. Movie was processed every 114 minutes for size reduction. Total imaging time = 85 hours. Playback speed is 38,250×real time.
(AVI)

Movie S2 **Time-lapse observation of cells with mAG^+^ and mKO2^+^ nuclei in a draining LN in a #639/#474 mouse.** Movie was processed from the same observation area of **Fig. 2e**. A region was time-lapse imaged with the z step size of 5 µm every 30 sec for 30 min. Z stacked images (10 µm thick) are shown in this movie.
(MOV)

Movie S3 **Time-lapse observation of cells with mAG^+^ and mKO2^+^ nuclei in a draining LN in a #639/#474 mouse.** Movie was processed from the same observation area of **Fig. 2f**. A region was time-lapse imaged with the z step size of 5 µm every 30 sec for 30 min. Z stacked images (10 µm thick) are shown in this movie.
(MOV)
